# Mesothelial Cyst of the Round Ligament Misdiagnosed as Irreducible Inguinal Hernia

**DOI:** 10.1155/2013/408078

**Published:** 2013-09-18

**Authors:** Dimitrios K. Manatakis, Nikolaos Stamos, Christos Agalianos, Panagiotis Vamvakas, Athanasios Kordelas, Demetrios Davides

**Affiliations:** ^1^1st Surgical Department, Athens Naval and Veterans Hospital, 70 Dinokratous street, 11521 Athens, Greece; ^2^Department of Pathology, Athens Naval and Veterans Hospital, 70 Deinokratous Street, 11521 Athens, Greece

## Abstract

We report the case of a 36-year-old woman who presented with signs and symptoms of an irreducible inguinal hernia. Surgical exploration revealed a mesothelial cyst of the round ligament of the uterus. Mesothelial cysts of the round ligament are rare lesions, frequently masquerading as inguinal hernias, and should be included in the differential diagnosis of any inguinal mass. Clinical findings are those of a groin mass, discomfort, and bulging. Ultrasound and CT scans often demonstrate an aperistaltic cystic mass. Definitive diagnosis is usually made intraoperatively and confirmed histopathologically.

## 1. Introduction

 Differential diagnosis of a groin mass includes inguinal and femoral hernias, lymphadenopathy, benign or malignant tumours, saphenous vein varicosities, femoral artery aneurysms, abscesses, dermoid, sebaceous and pilonidal cysts and cystic lymphangiomas. Sex-specific pathologies include an undescended testis or a hydrocele of the spermatic cord in males and cysts, varicosities and endometriosis of the round ligament or herniation of the ovary in females [1].

We present and discuss a case of a rare mesothelial cyst of the uterine round ligament misdiagnosed as irreducible inguinal hernia and review the relevant literature.

## 2. Case Presentation

 A 36-year-old, female, caucasian, and multiparous patient presented at the outpatient surgical clinic with a twelve-month history of a right inguinal bulge and discomfort, with progressive worsening of pain over the past 24 hours. Past medical and surgical history was unremarkable. 

 On clinical examination, a relatively firm, smooth inguinal mass was revealed, roughly 4 × 2 cm, medial to Poupart's ligament, tender to palpation, and protruding when the Valsalva manoeuver was performed. Bowel movements were normal, without vomiting, abdominal distention, or signs of intestinal obstruction. Laboratory tests, and plain abdominal radiographs were within normal range. A preoperative diagnosis of an irreducible right inguinal hernia was made, and the patient consented to surgical treatment. 

**Figure 1:**
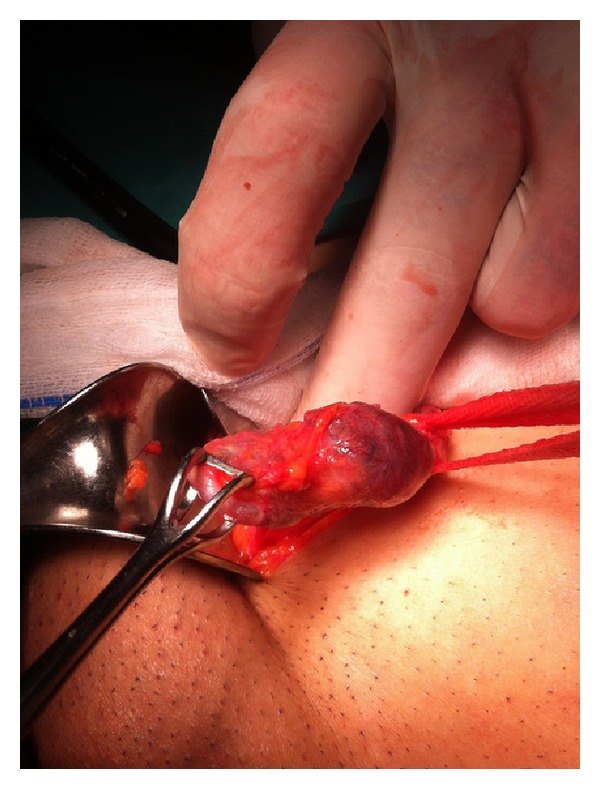
Intraoperative photograph of the mesothelial cyst originating from the round ligament.

 Under general anaesthesia, the right groin was explored. The superficial inguinal ring appeared normal. Following incision of the external oblique aponeurosis, the round ligament was found unusually thin, and a multilobular clear-fluid cystic lesion, approximately 4 cm in diameter, originating from the round ligament, was observed (Figure 1). The lesion was dissected and excised. A concurrent small direct hernia was repaired by the plug-and-patch tension-free technique. The deep inguinal ring was found normal on examination, without protrusion of an indirect hernia sac. 

**Figure 2:**
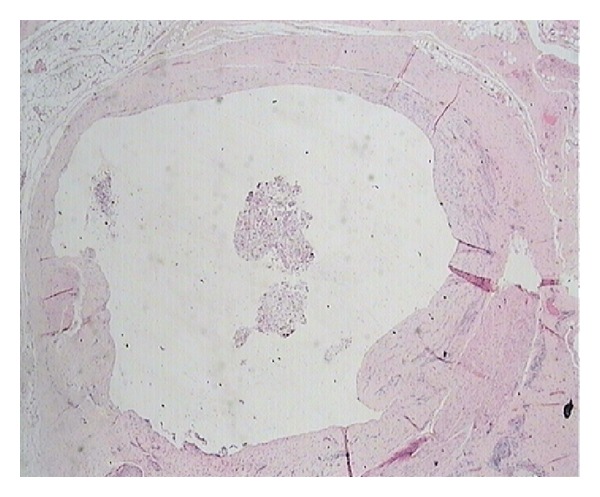
Apoptosis of papillae into the cyst lumen (H&E ×20).

**Figure 3:**
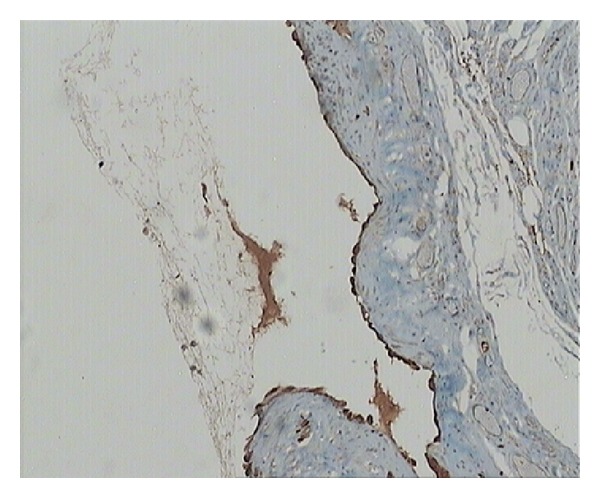
Calretinin stain (IHC ×100).

**Figure 4:**
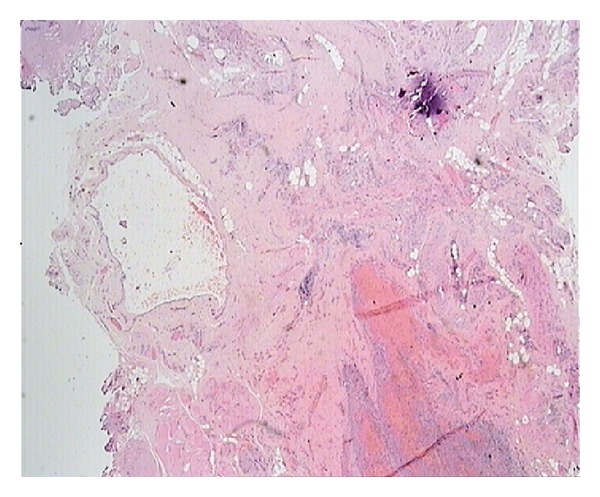
Endometriotic tissue (lower right) adjacent to the cystic wall (H&E ×20).

Histopathology demonstrated a multilobular cyst of 3 × 3 × 2 cm, filled with seromucinous clear whitish fluid, lined with a single layer of cuboidal cells, with focal areas of papillary architecture and apoptosis of papillae into the lumen (Figure 2). Immunohistochemistry was positive for pankeratin and calretinin, confirming mesothelial origin (Figure 3). The cystic wall was composed of dense fibrous tissue, smooth muscle fibers, and vessels. A ruptured endometriotic cyst, adjacent to the mesothelial cyst, was also found in the specimen (Figure 4).

 Patient's recovery was uneventful. She was discharged 24 hours postoperatively and was followed up for 6 months, without recurrence or further symptoms. 

## 3. Discussion

 Mesothelial cysts of the round ligament of the uterus are a rare pathology. A search of the English-speaking literature between 1980 and 2013 revealed only 10 cases. They are usually misdiagnosed as inguinal hernias and detected accidentally during groin exploration [2–6]. They are often associated with small, clinically insignificant inguinal hernias in 30–50% of cases [2–4]. Most patients are in their late thirties and forties [2]. Of interest, a right-side preponderance has been observed without any plausible explanation given in the literature [2, 7].

 Mesothelial cysts are generally considered developmental disorders. There are two proposed theories of pathogenesis based on the embryology of the round ligament and the histologic appearance of the lesions [2, 4–6]. 

 At the 7th week of gestation, the fetal inguinal fold differentiates. The gubernaculum develops and descends from the lower pole of the gonads to the labioscrotal swelling. At the 12th week, the ovary descends to the pelvic rim and the cephalad half of the gubernaculum fuses to the uterus at the position of the uterine tubes. The caudal half forms the round ligament. During its descend along with a portion of the peritoneum called processus vaginalis or Nuck's canal in women, elements of layers of the abdominal wall are incorporated in the round ligament. Thus, the fully developed round ligament consists mainly of smooth muscle fibers looped together in bundles, separated by fibrous tissue septa and containing blood vessels and nerve fibers, within a mesothelial investment [2]. 

 The first theory is based on a flawed obliteration of Nuck's canal. Depending on the level of the flaw, cysts may form at any point along the round ligament and can be pedunculated or wide-based. According to this theory a mesothelial cyst is the same disease as a cyst of the canal of Nuck. A comparable flaw in males leads to formation of hydrocele of the spermatic cord, communicating or not [2, 8]. 

 The second theory attributes cyst formation to the inclusion of embryonic, mesenchymal, and mesothelial elements or remnants, during the development of the round ligament. In support of this mechanism, Harper Jr. et al. documented small mesothelial cystic inclusions within the body of the round ligament [2].

 A third speculation would be the de novo development of the cyst, much like the development of benign cystic mesothelioma (BCM) arising from mesothelium-lined serosal surfaces, with which mesothelial cyst shares common histopathological characteristics [9, 10]. BCM is controversially regarded as either reactive or neoplastic [9, 11]. An association has been reported with past abdominal surgery, intraabdominal pelvic inflammation, or endometriosis, as might have been the case with our patient. However, other authors accept its neoplastic nature and malignant potential [12, 13].

 Establishing an accurate preoperative diagnosis, based solely on clinical features, is challenging [2]. Mesothelial cysts are usually asymptomatic or tend to produce subtler symptoms compared to hernias [3, 8]. Pain, discomfort, or sensation of heaviness and bulging are the predominant complaints [2]. In the absence of a concurrent hernia, cysts should not change in size with the Valsalva manoeuver. If the inguinal mass bulges with Valsalva and disappears in the supine position, a hernia is a more probable diagnosis. During pregnancy, hernias tend to disappear and no strangulation is expected, because the bowel is pushed by the enlarged uterus [14]. Varicosities of the round ligament on the other hand worsen during pregnancy, because of elevated intraabdominal pressure, and should be included in the differential diagnosis [14–16]. 

 Preoperative diagnosis is greatly aided by imaging studies. Ultrasound is the modality of choice, harbouring no danger of radiation especially for children and young women, who consist the main prevalence population. It is a real-time examination, offering information about intestinal peristalsis, vascular supply, and changes in size with cough or Valsalva manoeuver. Various sonographic appearances have been described: comaform, fusiform, or oval cystic mass, with internal septa if multilobular and with no peristalsis. Pedunculate lesions are attached to a stalk-like structure, which is their connection to the peritoneal cavity. Occasionally, hypoechoic solid portions may be observed [4, 6]. 

 Computed tomography reveals a cystic mass with irregular wall thickening and enhancement of the solid parts with intravenous contrast medium. Attention should be paid not to misdiagnose the cyst as metastatic locus, when a primary neoplasm is discovered or when the patient has a history of malignancy [4]. 

 Magnetic resonance is a more expensive option, providing more detail of the adjacent anatomic structures, and it should be reserved for those cases that pose a diagnostic conundrum [17]. Findings consist of a thin-walled cyst, hypointense on T1-weighted images, and hyperintense on T2-weighted images. Internal septation, if present, appears hypointense [18, 19].

 Definitive diagnosis is made macroscopically at surgery and confirmed by histopathology. Our findings of a single layer of cuboidal cells lining the cyst and staining positive for calretinin and pankeratin are consistent with the literature [3, 20]. Although the lesion affects mostly women of reproductive age, it does not seem to be sex hormone-dependent. The perceived enlargement after gonadotropin stimulation for in vitro fertilization in the case report of Ryley et al. was attributed to changes in size of the round ligament and not the cyst itself, while the stain for estrogen and progesterone was negative [3]. 

 There is little evidence in the literature to safely recommend a treatment or follow-up protocol. Given the benign nature of the disorder, a reasonable option would be to observe the asymptomatic patient with serial ultrasound examinations. Cysts that become symptomatic or grow in size over time are better treated by surgical excision [3]. Aspiration of the cyst under ultrasound guidance has also been described, and offers temporary relief from pain, but quickly leads to fluid reaccumulation [8]. Following excision prognosis is excellent, and no recurrences have been reported. 

## 4. Conclusion

Mesothelial cyst of the round ligament of the uterus is a rare pathology, which should be included in the differential diagnosis of groin masses in women. It is frequently misdiagnosed as inguinal hernia, since clinical findings are those of an irreducible inguinal mass, discomfort, and bulging. Ultrasound and CT scan may help with the diagnostic workup, but definitive diagnosis is usually made intraoperatively. Surgical excision relieves symptoms, and histopathology confirms the nature of the lesion.
